# Foreign patients in emergency departments of Japanese medical facilities

**DOI:** 10.1002/ams2.781

**Published:** 2022-09-07

**Authors:** Soichiro Saeki, Koichiro Tomiyama

**Affiliations:** ^1^ Department of Emergency Medicine and Critical Care Center Hospital of the National Center for Global Health and Medicine Tokyo Japan

**Keywords:** Foreign residents, global health, health‐care interpreters, medical interpreters, non‐national patients

## Abstract

Our manuscript is a letter to the article “Factors associated with emergency department length of stay of foreign patients visiting a regional core hospital in Japan.” by Aoki *et al*. published ahead of print in Acute Medicine and Surgery. We believe that the manuscript by Aoki *et al*. is the first publication to suggest that linguistics play an important role in the outcomes of foreign patients in Japanese medical facilities, and applaud them for their contribution. However, we also believe that further assessments are required to make their implications more robust. Therefore, in our manuscript, we would like to highlight several criteria that should be included in such studies, derived from studies that have been conducted under similar situations in Japanese medical facilities.

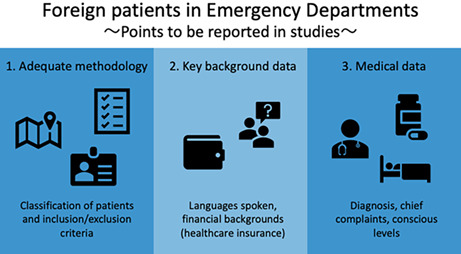

Dear Editor,

We applaud Aoki *et al*.^1^ and their colleagues in Aizawa Hospital for their fine achievement in *Acute Medicine and Surgery*. Their findings are one of the novel manuscripts to suggest that language influences emergency department length of stay from live data from a clinical setting. As Japan has promoted linguistic assistance for foreign patients as a nation,^2^ this article should further encourage the government to continue such efforts.

However, as such data on foreign patients in Japan are still lacking,^2^, ^3^ we also hope that related research would continue to be shared from medical facilities across Japan. Therefore, we hereby wish to highlight several points that should be clarified when similar research would be published.

First, the methods for obtaining and categorizing data on foreign patients should be specified. For instance, how the background data of patients were gathered should be indicated. In a clinical setting, it is difficult to determine a patient's nationality or status of stay.^4^ Although this manuscript defined residents as patients living in Japan for 3 consecutive months, patients with residential visas may visit a hospital as soon as they arrived. To replicate such studies elsewhere, we believe that such definitions should be outlined, along with how exactly the patients were classified into such definitions.

Second, additional key data regarding the patients and study settings should be reported. One, the criteria for using medical interpreters should be reported. This is important to assess the extent to which linguistic communication is achieved with each patient. Two, information about the patient's insurance coverage, which is relatively easy to obtain in many hospitals,^4^ should be stated. Financial backgrounds may influence the attitudes of patients toward the amount of medical care they want to receive, and such data would also aid the understanding of the patient's backgrounds.^4^

Finally, as the study design is in an emergency department, further information on the patient condition should also be provided. As in previous related studies,^4^, ^5^ data on the patient's diagnosis would be of further assistance in assessing the results in detail, especially classified along with the key resulting factor; in this case, language. It would also be beneficial to report the chief complaints of the patients. As the authors stated,^1^ consultations for injuries are typically simple, requiring less time and effort compared with other symptoms such as hyperthermia. In addition, data on the consciousness level, such as the Glasgow Coma Scale (GCS) score, may be useful. A previous study^5^ found that altered mental status hampered communication with patients, potentially leading to an increase in consultation time.

Overall, we are in consensus with the authors^1^ that linguistics is vital in medical contexts and that language aid should be encouraged. However, their current results may not be sufficient to strongly support their conclusion, and additional research is required to strengthen the generalizability of their findings. We hope that methods of obtaining data from patients would become more standardized so that further data from other facilities may be compared and evaluated across Japan.

## Funding information

No funding information provided.

## DISCLOSURE

Conflict of interest: None declared.
